# Calcium intake may explain the reduction of colorectal cancer odds by dietary selenium - a case-control study in Poland

**DOI:** 10.1186/s40795-022-00515-w

**Published:** 2022-03-14

**Authors:** Malgorzata Augustyniak, Aleksander Galas

**Affiliations:** Chair of Epidemiology and Preventive Medicine, Department of Epidemiology, Jagiellonian University Medical College, 7 Kopernika St, 31034 Krakow, Poland

**Keywords:** Dietary selenium, Dietary calcium, Large bowel cancer, Risk estimate, Modification effect

## Abstract

**Background:**

Colorectal cancer (CRC) has been placed among top three cancer sites in high income countries. Although there are several inconsistencies across studies it is widely accepted that diet contributes to approximately 70% of CRC. Several dietary factors have been investigated; however, the knowledge about the role of trace elements and their interplay with other dietary factors in CRC odds is limited. The aim of the study was to estimate the odds ratio of colorectal cancer associated with the content of selenium in diet, and to check whether dietary calcium is a modifier of selenium effect in the population characterized by low selenium intake.

**Methods:**

Face-to-face interviews were used to gather data on dietary habits (by 148-item semi-quantitative food frequency questionnaire) and covariates among 683 histologically confirmed incident colorectal cancer cases and 759 hospital-based controls in a case-control study. Data was collected in a period between 2000 and 2012. Setting: Lesser Poland, Central Europe. Logistic regression models were used to assess the role of dietary selenium intake and calcium-selenium interaction in colorectal cancer odds.

**Results:**

After the adjustment for several covariates dietary selenium was associated with the decrease of colorectal cancer odds by 8% (OR = 0.92, 95%CI: 0.84–0.99 for every 10μg Se/day increase). In individuals with lower (< 1000 mg/day) calcium content the odds of colorectal cancer was decreased by 13%(for every 10μg Se/day) and by 44% and 66% depending on the categories of selenium intake (60 to < 80 μg/day and ≥ 80 μg/day, respectively). The effect of dietary selenium was modified by dietary calcium (*p* for interaction < .005).

**Conclusions:**

The study has shown a beneficial effect of dietary selenium for colorectal cancer and a modification effect of dietary calcium in a population characterized by lower levels of selenium intake. The results provide the basis for well-planned controlled trials to confirm the findings.

**Supplementary Information:**

The online version contains supplementary material available at 10.1186/s40795-022-00515-w.

## Background

Colorectal cancer (CRC) has been placed among top three cancer sites representing morbidity and mortality in high income countries for several years, even if there are numerous programs and policies which target that issue. Cancer burden is responsible for approximately 1.9 million cases and consequently almost 1 million deaths worldwide [1]. Out of these, CRC contributes to approximately 10% of new cases and 9.2% of cancer deaths [1]. The descriptive epidemiology clearly shows the need for effective prevention strategies, but, although there are several known factors which contribute to the CRC risk, the evidence about their role is not consistent. The World Cancer Research Fund and the American Institute for Cancer Research in their Continuous Update Project, which analysed research on cancer prevention and survival, mentioned physical activity only as a factor which decreases the CRC risk, and for which the evidence is strong and convincing. On the other hand, processed meat, alcoholic drinks, body fatness were identified as risk factors with convincing, strong evidence [2]. Although there are several inconsistencies across different study results which tried to assess the impact of different factors associated with colorectal cancer, it is somehow accepted that approximately 70% of CRC are caused by behavioural factors including diet [3].

An essential trace element found in brazil nuts, fish, ham, meat, cereals and pasta [4], selenium, is among dietary factors which are in focus. The element may prevent against cancer by affecting the expression of gene Bcl-2 and p53 [5, 6], and as a part of selenoproteins by the enhancement of reactive oxygen species elimination, and a down regulation of the interleukin-2 [7], impact on intracellular signalling [8], activation of thyroid hormone and enhanced apoptosis [9, 10]. Selenium, therefore, has been investigated across different studies in humans, however, with different results. There are observational studies performed in Australia [11], among US Whites and US African Americans [12], and an intervention study in the eastern US [13] which showed protective effect against CRC. There are also studies, and even metaanalyses of controlled trials, which results did not reach statistical significance [14, 15]. The inconsistencies observed may be caused by several factors including: 1) distorting effects caused by several possible covariates, which were not considered in the performed analyses, and 2) a variability of exposure levels, meaning levels of selenium intake, considered by different authors; especially if there is a possibility of the U-shaped effect between selenium intake and cancer risks [16], and additionally 3) a possibility of a composite effect of selenium with other dietary compounds.

The purpose of the presented study was to estimate the odds ratio of colorectal cancer associated with a content of selenium in diet and to check whether other dietary item as calcium has an impact on the selenium effect. In details, we have analysed whether dietary selenium overall, as well as a consumption of higher than 60 μg/day dietary selenium, after adjustment for key covariates, decreased the CRC odds ratio in Lesser Poland, central Europe, characterized by relatively lower selenium intake. Moreover, dietary calcium and selenium have different points of action. Dietary calcium is involved in colon epithelial cell protection by a formation of calcium-phosphate-bile acid complexes, and at a cell level takes part in the cell differentiation and growth. Selenium is an active compound of glutathione peroxidases which prevents a formation of reactive oxygen-centered radicals (antioxidant function), and additionally takes part in the control of transcription factors, proliferation and apoptosis. Therefore, the two factors may interplay leading to CRC odds which differs from the odds expected to result from their individual effects. Considering this, an interaction between dietary selenium and dietary calcium was additionally analysed, and the colorectal cancer odds ratio across different subgroups characterized by different selenium and calcium intake levels has been investigated.

## Material and methods

### Study population

**Fig. 1:**
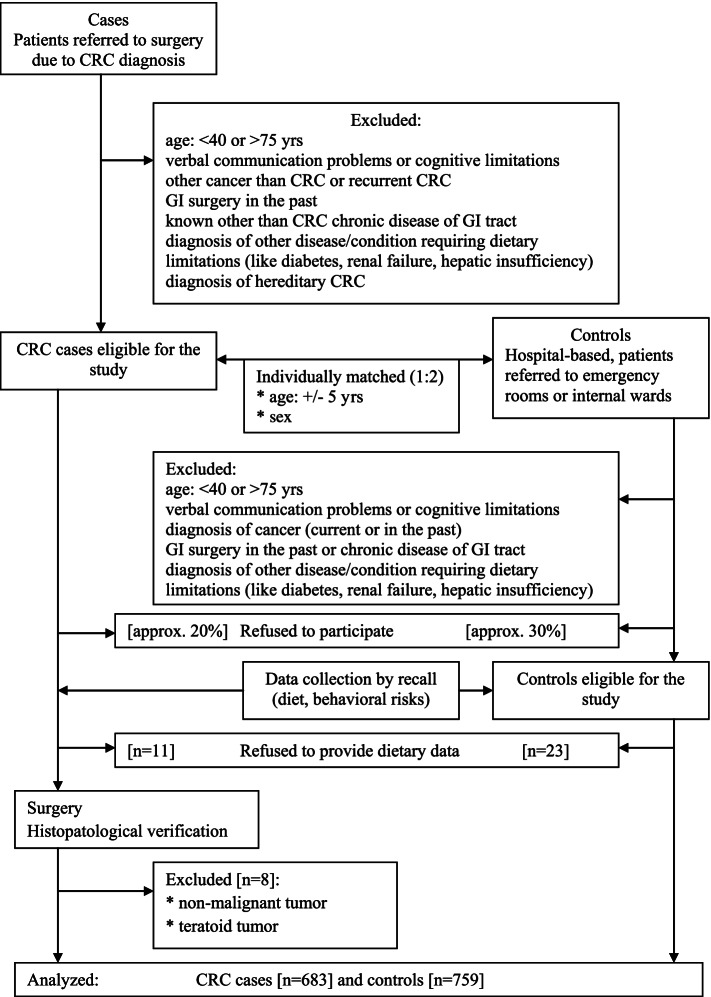
The study flow

The current study is based on a hospital-based case-control study which was performed during 2000–2012. The design of the study, data collection methods, and primary purposes have been described elsewhere [17]. In brief, only incident cases of CRC were identified and recruited in the cooperation with the [blinded for the review]. Those should be histologically confirmed adenocarcinomas either of colon (ICD-X: C18) or rectum (ICD-X: C20). Only sporadic cancers met eligibility criteria, meaning all CRC cases suspected to be hereditary cancers were excluded (ICD-X: C18.9, D12.6, Q85.8, Z80.0, Z80.3). Additionally, to limit the possibility of genetically determined CRC for the current study, cases younger than 40 years of age were also excluded. Controls were patients admitted to emergency rooms and next hospitalized in the University Hospital in Krakow and the Narutowicz Municipal Hospital in Krakow, Poland. Primarily, the controls were individually matched to cases on age (range +/− 5 years) and sex with the frequency ratio 1:2 meaning we tried to identify two controls for a case being the same sex and at similar age. The study flow is presented on Fig. 1. Among exclusion criteria for both, cases and controls, were: age over 75, cognitive limitations and verbal communication problems which caused reviewers could not proceed a recall, a diagnosis of secondary cancer, or CRC being a metastasis in colon or rectum, and any other than CRC cancer (current or in the past), any type surgery of gastrointestinal tract in the past, current or past diagnosis of chronic gastrointestinal disease, and any other disease requiring dietary limitations (like diabetes, renal failure, hepatic insufficiency), and additionally a presence of prolonged (lasting longer than a month) gastrointestinal symptoms were verified.

The study was conducted according to the guidelines laid down in the Declaration of Helsinki and all procedures involving research study participants were approved by the Jagiellonian University Ethical Committee (IRB no 1072.6120.347.2020). Written informed consent was obtained from each study participant.

### Data collection and assessment

The data used for the presented study was gathered by standardized questionnaire. Trained interviewers collected information about socio-demographic characteristic, lifetime smoking habits, lifetime physical activity and diet during face-to-face recall. In details, a semi-quantitative validated food frequency questionnaire prepared in the cooperation with the German Cancer Research Centre, Heidelberg, Germany, during the preparation phase for the European Prospective Investigation into Cancer and Nutrition study [18, 19] was implemented to assess dietary habits. In total, 148-dietary items (food and drinks), were used. There were questions about consumption of cereals, dairy products, bread, type and cuts of meat and fish, (including preparation technique), fresh fruit (during summer and winter time separately), salads, fresh and cooked vegetables, rice and pasta, soups, sweets, baked goods, and others. Study participants estimated commonly consumed portion size, for each food and beverage, using standardized photographs, and next, a frequency of consumption was reported. Dietary data covered the period of 1 calendar year. Case-participants were questioned about the year which took place 5 years prior to the onset of gastrointestinal symptoms (if symptoms were present) or prior to the beginning of a diagnostic process. Control patients reported their usual diet which took place 5 years before the interview. Next, to get data on macro and micronutrient intake, the Polish food composition tables were used. As the content of selenium was not available in the primary tables, for the current study information about intake of each dietary item was additionally used to calculate the content of dietary selenium. The concentrations of selenium, including losses on cooking, for each dietary item assessed, have been presented in the additional materials (see Supplementary materials).

### Covariates

Several covariates which might contribute to colorectal cancer odds have been collected. These included adult lifetime leisure time physical activity, which was assessed by a recall as the average weekly time spent on different type activities (including walking or hiking, bicycling, gardening, practicing sports and household activities) during summer and winter season separately. Additionally, respondents were asked about amount of time spent on recreational activities requiring at least moderate effort. Next, to get metabolic equivalents, the reported time for each activity was multiplied by its typical energy expenditure requirement as published in the 2011 Compendium of Physical Activities [20]. Considering participants were not asked about brakes in their activities, it was assumed that 70% of reported time was spent effectively and these was presented and used as a covariate in analyses. Among other covariates respondent’s age, sex, body mass index calculated from the weight and height measured at the admission to a clinic or hospital, the exposure to cigarette smoke (categorized as non-smoker, past smoker, current smoker) were considered. Moreover, the set of dietary covariates were used, and these included total dietary fibres, intake of dietary vitamin c and vitamin e, fish consumption (categorized yes/no; “yes” for participants reported consumption of any fish, having a portion size of at least 20 g of canned fish or 45 g of cooked fish, monthly), and taking mineral supplements (categorized yes/no).

### Statistical analysis

There were two groups included in study analyses, colorectal cancer cases and controls, presented and compared in order to assess the role of dietary selenium. The groups were characterized provided means and standard deviation, and additionally, as majority of variables had skewed distributions (tested by the Shapiro-Wilk test of normality), median and interquartile range was provided in the descriptive part. The study groups were compared using the chi-squared test for categorized variables (all the expected cell values in the analyses fulfilled the assumption of being over 5), and the U-Mann-Whitney test (as the variable distribution in groups compared were skewed). To assess the role of dietary selenium intake on the CRC odds the logistic regression was used. We have decided to run 3 consecutive models: univariable one, next multivariable adjusted for the main confounders as age, sex, body mass index (BMI), average adult lifetime leisure-time physical activity, and alcohol consumption, and smoking. The third model additionally included as covariates main dietary components as total dietary fibres, dietary content of vitamin c and vitamin e, taking mineral supplements, dietary calcium intake, and fish consumption. There were models to assess odds associated with selenium considered as a continuous variable (results presented for an increase by 10 μg/day of dietary selenium) and also across categories of selenium intake (< 60 μg/day – the reference group; 60 to < 80 μg/day and ≥ 80 μg/day). We have decided to use all three models to verify a stability of selenium effect estimates. Finally, to verify the presence of an additional role of calcium, all regression analyses have been repeated across lower (< 1000 mg/day) and higher (≥1000 mg/day) dietary calcium intake subgroups. And, as we observed effects of selenium which varied across different dietary calcium intake levels, the last part of the analyses done assessed the odds of CRC across different categories created by both, the consumption of selenium and consumption of calcium. There were 9 categories created by the cut-offs of 60 μg/day and 80 μg/day of selenium and 1000 mg/day and 1500 mg/day of calcium. Subsequently the logistic regression model adjusted for all considered covariates with the reference category of < 60 μg/day of selenium and < 1000 mg/day of calcium was run. This had been done to show a possible modification effect of calcium in the selenium effect. Missing data has been reported in the descriptive part, and the pair-wise procedure was used in the multivariable analyses.

## Results

There were 683 CRC cases and 759 controls eligible for analyses in the presented study. Controls were mainly represented by patients diagnosed with disorders of thyroid gland (E00-E07: *N* = 20, 2.6%), hypertensive diseases (I10-I15: *N* = 96, 12.6%), ischemic heart disease (I20-I24: *N* = 119, 15.7%), and other forms of heart disease (I30-I52: *N* = 33, 4.3%), diseases of respiratory system (J00-J99: *N* = 65, 8.6%), hernia (K40-K46: *N* = 79, 10.4%), disorders of gallbladder (K80-K87: *N* = 143, 18.8%), syncope and collapse (R55: *N* = 18, 2.4%), injuries (S00-S99: *N* = 34, 4.5%), and others (see Additional file 1: Appendix 2 for details).

The characteristic of the study participants has been presented in Table 1. Among significant differences observed between groups were: sex distribution, as there were more men in the CRC group (57% vs. 52%) and age distribution (CRC were slightly older, medians: 59 yrs. vs. 57 yrs.). Simple comparison of dietary items in investigated groups showed differences in the intake of calcium (CRC vs. controls, medians: 652 mg/day vs. 688 mg/day) and consumption of alcohol (pure ethanol, recalculated, medians: 8.2 g/week vs. 4.8 g/week).

**Table 1:** Basic characteristic of study participants

	Colorectal cancer cases [*n* = 683]		Controls [*n* = 759]		*p*-value
Gender [n, (%)]					p^chi2^ = .049
Men	389	(57.0%)	393	(51.8%)	
Women	294	(43.0%)	366	(48.2%)	
Age					p^MW^ = .010
mean, (SD)	58.6	(8.0) ^$^	57.5	(9.0) ^$^	
median (Q1-Q3)	59.0	(53.0–65.0)	57.0	(50.0–65.0)	
Dukes	[*n* = 464]				
A (T1, N0, M0)	32	(6.9%)			
B (T2, N0, M0)	100	(21.6%)			
B2 (T3–4, N0, M0)	97	(20.9%)			
C1 (T2, N1–3, M0)	24	(5.2%)			
C2 (T3–4, N1–3, M0)	98	(21.1%)			
D (Tx-4, Nx-3, M1)	113	(24.4%)			
Histological grade	[*n* = 129]				
Grade 1 (G1)	31	(24.0%)			
Grade 2 (G2)	87	(67.4%)			
Grade 3 (G3)	11	(8.5%)			
Leisure-time physical activity* [METs-h/week]			[*n* = 757]		p^MW^ = .301
mean, (SD)	31.5	(26.4) ^$^	31.4	(22.5) ^$^	
median (Q1-Q3)	26.6	(11.6–43.9)	28.3	(13.6–43.8)	
BMI [kg/m2]			[n = 757]		p^MW^ = .637
mean, (SD)	27.4	(4.2)^$^	27.6	(9.9) ^$^	
median (Q1-Q3)	27.0	(24.5–29.7)	27.0	(24.2–29.9)	
Smoking [n, (%)]	[*n* = 557]		[*n* = 675]		p^chi2^ = .073
Current smoker	149	(26.8%)	220	(32.6%)	
Past smoker	164	(29.4%)	175	(25.9%)	
Non-smoker	244	(43.8%)	280	(41.5%)	
Dietary calcium [mg/d]					p^MW^ = .035
mean (SD)	712	(299) ^$^	783	(419) ^$^	
median (Q1-Q3)	652	(496–856)	688	(491–929)	
Dietary selenium [μg/d]					p^MW^ = .304
mean (SD)	51	(17) ^$^	55	(27) ^$^	
median (Q1-Q3)	49	(39–59)	50	(38–64)	
Total dietary fibre [g/day]					p^MW^ = .718
mean (SD)	17.9	(6.5) ^$^	18.5	(7.8) ^$^	
median (Q1-Q3)	16.8	(13.1–21.4)	16.8	(12.9–22.0)	
Pure alcohol** [g/week]					p^MW^ = .004
mean (SD)	33.5	(67.7) ^$^	30.9	(78.6) ^$^	
median (Q1-Q3)	8.2	(2.8–32.2)	4.8	(2.4–25.5)	
Vitamin C [mg/d]					p^MW^ = .381
mean (SD)	93	(51) ^$^	99	(62) ^$^	
median (Q1-Q3)	78	(58–117)	82	(55–132)	
Vitamin E [mg/d]					p^MW^ = .860
mean (SD)	9.8	(4.8) ^$^	10.2	(5.3) ^$^	
median (Q1-Q3)	8.8	(6.7–11.5)	8.7	(6.4–12.6)	
Taking mineral supplements [n, (%)]	102	(14.9%)	111	(14.6%)	p^chi2^ = .869
Fish consumption [n, (%)]					p^chi2^ = .891
No	42	(6.2%)	48	(6.3%)	
Yes	641	(93.8%)	711	(93.7%)	

Continuous variables were presented as mean (SD) or median (Q1-Q3) and *P* values were calculated using the Mann-Whitney test; categorical variables were presented as n (%) and *P* values were calculated using chi-square test

*BMI* Body mass index, *Chi2* Chi-squared test, *MW* the Mann-Whitney test

^$^*p*-value <.05 by the Shapiro-Wilk test for normal distribution

^*^average adult lifetime physical activity (reported as recreational and at home)

^**^participants reported their consumption expressed in “standard” drinks, which is an equivalent of 12 fl of regular beer of about 5% alcohol or 5 fl of wine (approximately 12% alcohol) or 1.5 fl of distilled spirits (gin, rum, vodka, whiskey, 40% alcohol). Each “standard” drink contains roughly 14 g of pure alcohol

The main study question was about the role of selenium in CRC. The estimated effect of dietary selenium intake (meaning the increase in diet by each 10 μg/day) has been associated with a decrease in the odds ratio by 10 to 6%, depending on the number of covariates considered (Table 2). The subgroup analyses in lower (< 1000 mg/day) and higher (≥1000 mg/day) contents of dietary calcium revealed that selenium decreased the odds ratio in the fully adjusted model among those who had diet with lower calcium content (OR = 0.87, 95%CI: 0.78–0.98). The effect of selenium, however, was not observed as statistically significant in the group of high calcium intake. Additionally, we have tested whether the selenium effect persisted provided the categories of selenium intake were considered. We observed a decrease in the CRC odds ratio across the selenium categories by approximately 30 and 60% in the 60 to < 80 μg/day, and ≥ 80 μg/day, respectively (Table 2). The primary models considered calcium as a covariate. Next, analyses across calcium intake levels were done, and the effect of selenium was observed in the lower calcium intake (odds ratio reduction by 44 and 66% across selenium categories, respectively), but it was not typically noticed in the higher calcium intake subgroup. As we hypothesized that effect of selenium was dependent on the level of calcium intake, we run two models which tested interaction term between selenium and calcium. Both, a model which considered selenium and calcium only, and a model with all considered covariates showed statistically significant interaction (*p* = .001 and *p* = .002, respectively). Therefore, as a last part of the analysis we run a model in which CRC odds ratio was estimated across 9 groups of patients categorized on the basis of their selenium (3 categories: < 60 μg/day, 60 to < 80 μg/day, and ≥ 80 μg/day) and calcium (3 categories: < 1000 mg/day, 1000 to < 1500 mg/day, and ≥ 1500 mg/day) dietary intake. Fully adjusted logistic regression model showed interesting results, as there was not significant effect observed with an increase of calcium consumption provided individuals consumed small, i.e. < 60 μg/day selenium. Increase in the selenium intake decreased the odds ratio of CRC among individuals with low (< 1000 mg/day) intake of calcium, but among higher categories of calcium intake selenium had more visible impact if its consumption was clearly higher (≥80 μg/day). And finally, the lowest CRC odds ratio was observed for those study participants who were categorized into the group characterized by both, the highest selenium and the highest calcium intake levels (OR = 0.09, 95% CI: 0.03–0.33) (Table 3).

**Table 2:** Selenium dietary intake and odds ratios for colorectal cancer (overall, and by strata of dietary calcium intake)

	Model 1			Model 2			Model 3		
	OR	95% CI	*p*-value	OR	95% CI	*p*-value	OR	95% CI	*p*-value
Dietary selenium intake (continuous)									
for and each increase by 10 μg/day	0.934	0.889–0.980	.006	0.892	0.839–0.948	<.001	0.918	0.843–0.999	0.048
Dietary selenium intake (categorized, reference group < 60 μg/day)									
60 to < 80 μg/day	0.814	0.619–1.070	.140	0.699	0.516–0.947	.021	0.717	0.513–1.002	.051
≥ 80 μg/day	0.462	0.318–0.672	<.001	0.396	0.258–0.608	<.001	0.419	0.247–0.712	.001
	*p*-value for trend = .005			*p*-value for trend < .001			*p*-value for trend = .001		
Dietary calcium < 1000 mg/day									
Dietary selenium intake (continuous)									
for and each increase by 10 μg/day	0.997	0.930–1.069	.935	0.927	0.852–1.010	.082	0.874	0.779–0.980	.021
Dietary selenium intake (categorized, reference group < 60 μg/day)									
60 to < 80 μg/day	0.809	0.585–1.121	.203	0.655	0.455–0.941	.022	0.562	0.378–0.835	.004
≥ 80 μg/day	0.574	0.326–1.011	.055	0.457	0.241–0.868	.017	0.332	0.162–0.680	.003
	*p*-value for trend = .066			*p*-value for trend = .002			*p*-value for trend < .001		
Dietary calcium ≥1000 mg/day									
Dietary selenium intake (continuous)									
for and each increase by 10 μg/day	0.894	0.806–0.992	.034	0.864	0.764–0.977	.020	0.955	0.851–1.072	.437
Dietary selenium intake (categorized, reference group < 60 μg/day)									
60 to < 80 μg/day	1.039	0.555–1.944	.905	0.844	0.424–1.679	.629	1.008	0.479–2.119	.984
≥ 80 μg/day	0.521	0.271–1.000	.050	0.475	0.230–0.980	.044	0.921	0.364–2.327	.862
	*p*-value for trend = .598			*p*-value for trend = .045			*p*-value for trend = .875		

Model 1 –univariable logistic regression

Model 2 –adjusted for age [years], sex, BMI [kg/m^2^], pure alcohol consumption [g/week], average adult lifetime leisure-time physical activity [METs-h/week], smoking (non-smoker, past smoker, current smoker)

Model 3 –adjusted for covariates used in Model 2 and additionally fish consumption [yes/no], total dietary fibre [g/day], vitamin C [mg/day] and vitamin E [mg/day], taking mineral supplements [yes/no], and dietary calcium [mg/day]

**Table 3:** Dietary selenium-calcium interplay in the risk of colorectal cancer (CRC). Stratum specified odds ratios with 95% confidence intervals for multivariable logistic regression^a^ which includes a considered exposure (dietary selenium intake) and an effect modifier (dietary calcium intake)

Calcium Selenium	< 1000 mg/day	1000 to < 1500 mg/day	> = 1500 mg/day
< 60 μg/day	1 (ref.)	0.711 (0.408–1.237)	1.109 (0.306–4.016)
60 to < 80 μg/day	0.634 (0.435–0.923)	0.767 (0.433–1.358)	0.296 (0.091–0.959)
≥80 μg/day	0.419 (0.211–0.830)	0.452 (0.225–0.908)	0.091 (0.025–0.328)

^a^adjusted for age [years], sex, BMI [kg/m^2^], pure alcohol consumption [g/week], average adult lifetime leisure-time physical activity [METs-h/week], smoking (non smoker, past smoker, current smoker), fish consumption [yes/no], total dietary fibre [g/day], vitamin C [mg/day] and vitamin E [mg/day], taking mineral supplements [yes/no]

## Discussion

We have decided to evaluate the role of dietary selenium in the risk of CRC as there are several inconsistencies in the published results. Additionally, we have investigated the impact of selenium considering dietary calcium as a possible modifying factor. The results we observed have shown a beneficial role of dietary selenium which decreased the CRC odds ratio by approximately 10% across analysed statistical models. Our results are in line with those published by Kune G and co-authors [11]. These authors, however, considered higher doses of selenium, and they observed a decrease in the CRC odds ratio across 81–99 μg/day, 100–118 μg/day, and 119–145 μg/day intake categories with no effect among higher than 145 μg/day doses. One should notice some important limitations in that publication, as the authors didn’t do adjustments for physical activity, and additionally they did not consider selenium losses on cooking. It is hard to comment possible effect of physical activity, but if the losses accounted for 10–20% in their population, the selenium concentration ranges and the observed magnitude of the effect would be similar to ours. The protective effect of selenium has been observed also in the North Carolina Colon Cancer Study-Phase II by Williams [12], with the risk reduction estimate of 45% in the highest quartile of dietary selenium category after adjustment for key covariates as age, sex, education, BMI, family history of CRC, non-steroidal anti-inflammatory drug use, and total energy. The positive effect of selenium has been observed by other authors too [21, 22], but in some publications the effect was noticed only among those with concomitant high folate intake [23].

As mentioned under introduction, there are some published study results and meta-analyses which did not show the beneficial role of selenium. There is the Nutritional Prevention of Cancer Trial, which investigated the effect of selenium supplementation of 200 μg daily in a randomized, double-blinded, placebo-controlled trial. The study in the secondary analyses showed non-significant effect of selenium in CRC risk. The risk reduction point estimate showed, however, a decrease by 54%, and it is worth noting the selenium supplementation reduced total cancer incidence in that study [15]. The later analysis of the impact of selenium supplementation showed the beneficial effect for colorectal adenomas among participants with the lowest tertile of baseline selenium [24]. In the updated metaanalysis published in 2016 by Cai [14], which evaluated the effect of selenium (different studies with different exposures as diet, supplementation, concentration in serum, in toenail), showed no significant effect; with moderate heterogeneity, without expectation of publication bias, with no impact of exposure mode, design type and study area.

We hypothesized the impact of the other factor which might interplay with dietary selenium. Our interest focused on dietary calcium. We considered that factor, as our previous observations showed the modifying effect of calcium when dietary fibre was analysed as a factor contributing to CRC odds ratio [17]. It is worth mentioning that we adjusted our models for fibre to minimize the possibility, that the observed selenium effect is a proxy of fibre.

It has been hypothesized the modification effect of calcium in the role of selenium as these two dietary elements have two different biological mechanisms which may explain their role. The main role of selenium in human body is that selenium is a part of an active centre of selenoproteins as selenocysteine. Some selenoproteins are linked to the risk of cancer, as glutathione peroxidases (GPxs) (a family of antioxidant enzymes), including GPx2 (gastrointestinal) which has an antiapoptotic function in colon crypts contributing to mucosal integrity [25], and additionally, which reduces peroxide in gut [26, 27], thioredoxin reductases (TrxRs), especially TrxR1 (cytoplasmic/nuclear) which takes part in the control of transcription factors, proliferation and apoptosis of cells [28], and selenoprotein S (SEPS1) with its anti-inflammatory properties [29]. Available research postulates calcium takes part in the formation of insoluble calcium-phosphate-bile acid complexes, thus decreases the concentration of soluble fatty acids. Luminal free fatty acids and bile fatty acids have been identified as factors which may damage epithelial cells in colon and may stimulate proliferation, increasing the risk of colon cancer [30]. At the cellular level calcium is postulated to promote cell differentiation and intracellular release of calcium restrains growth of colonic cells through calmodulin activation and promoting phosphorylation of intracellular enzymes [31].

To our knowledge, there are no published results which assessed the interplay between selenium and calcium in the CRC odds. In the selenium research, there are some publications which investigated the selenium – calcium interplay in the experimental animal models, which showed the normalization of Ca^2+^ leak caused by the leaky cardiac ryanodine receptor Ca^2+^ release channel (RyR2) after the treatment by sodium selenate, and additionally the normalization of an oxidative stress and Trx activity [32]. The need for selenoprotein for RyR2 channel activity had been also confirmed by other study [33]. Next, there are some published study results in polycyclic ovary syndrome patients which suggest selenium modulate neutrophil calcium channels and thus may provide a protective effect against oxidative stress and calcium concentration [34]. Therefore, considering several different biological mechanisms, and additionally some common points of action for both, selenium and calcium, we believe there is a possibility for a modification effect, although molecular pathways require to be investigated.

Our study, although representing a case-control design, has some worth mentioning benefits, as a relatively large sample size of the included CRC patients and controls, which increased the study power, and good quality information about diet, which had been collected by validated tool, and the use of approximately 150 dietary items. Additionally, the study design enables us collection of several data (covariates), which were subsequently used for adjustment, and in that way we were able to control for key demographic covariates, persons clinical characteristics, and dietary items. Adjustment for physical activity excluded a potential role of an established protective factor for CRC, next, the use of BMI enables us control for the long life energy intake-expenditure imbalance, and additionally to correct for a tissue which is a source of pro-inflammatory cytokines [35]. The observed stability of risk estimates decreases also the likelihood of random finding. Surely, the presented study is not free from limitations, as we did not consider the amount of selenium intake from supplements. In our study only general information about supplement use (yes/no) without details about supplement name or composition had been collected and used as a covariate. It is worth mentioning, however, that in other study performed in the US, the population in which the supplementation practices are more frequent, there was no special difference in the amount of selenium if supplements were considered or were not, and additionally, the “supplements did not provide additional risk reduction beyond intakes from food” [12].

## Conclusions

In summary, the presented study showed the beneficial effect of dietary selenium which decreased CRC odds ratio in the population characterized by low levels of intake. The effect of selenium has been modified by dietary calcium. Out study, therefore, provides some evidence for a reasonability of an increase of selenium and calcium intake in the subgroups characterized by low intake levels. Moreover, the results implicate the need for well-designed intervention studies which will consider both selenium and calcium statuses and intakes across intervention arms, and which will thus verify their protective effect and interplay.

## Supplementary Information

**Additional file 1: Appendix 1.** The concentration of selenium, including losses on cooking, by dietary item. **Appendix 2.** Controls’ diagnoses by ICD-X codes.

## Data Availability

The datasets used and analysed during the current study are available from the corresponding author on reasonable request.

## Abbreviations

BMI: Body mass index
CRC: Colorectal cancer
GPxs: Glutathione peroxidases
SEPS: Selenoprotein S
TrxR: Thioredoxin reductase
