# Supplementary material for: Calcium intake may explain the reduction of colorectal cancer odds by dietary selenium - a case-control study in Poland
Source: BMC Nutr. 2022 Mar 14;8:22. doi: 10.1186/s40795-022-00515-w (PMC8919630; doi:10.1186/s40795-022-00515-w)
Supplement: Supplementary file 1 — Additional file 1: Appendix 1. The concentration of selenium, including losses on cooking, by dietary item. Appendix 2. Controls’ diagnoses by ICD-X codes. [file 40795_2022_515_MOESM1_ESM.docx]

Appendix 1. The concentration of selenium, including losses on cooking, by dietary item

| Name of product | Selenium content in micrograms per 100g | Reference |
| --- | --- | --- |
| Cereal products and milk | | |
| Cornflakes | 2.6 | [1] |
| Milk and milk drinks | 1.7 | [2] |
| Breadstuff | | |
| White bread (rye, wheat) | 2.8 | [3] |
| Brown bread (wholemeal bread, graham bread) | 4.5 | [3] |
| Rolls (rye, wheat) | 3.5 | [3] |
| Brown rolls (i.e. graham rolls) | 3.9 | [3] |
| Other baker’s goods (i.e. French baguettes) | 27.1 | [4] |
| Spreadable fat products | | |
| Butter | 1.2 | [2] |
| Margarine | 5.3 | [5,6] |
| Vegetable butter | 3.3 | [5,6] |
| Cheeses and other bread additives | | |
| Cheese fresh | 4.5 | [1] |
| Cream cheese | 2.5 | [7,8] |
| Processed cheese | 2.0 | [8] |
| Cheese (i.e. Gouda, Edam, Emmentaler or other cheeses) | 3.0 | [7,8] |
| Blue cheeses (Camembert, Brie or other cheeses of this type) | 5.8 | [1] |
| Marmalade, jams, jellies | 10.7 | [1,5,8,9,10]* |
| Honey | 10.2 | [5,6] |
| Cold meats and fishes | | |
| Ham, loin, ham sausage, poultry ham and poultry loin | 7.2 | [5,6] |
| Salami and other dried sausages | 6.3 | [5,8] |
| Sausage i.e. ‘toruńska’ sausage; ‘litewska’ sausage; bacon (except for sausage intended for boiling) | 9.8 | [5] |
| Brawn, liver sausage, jellied cold meats | 18.2 | [5] |
| Rollmop, herrings in oil, fishes in tomato sauce | 14.6 | [11] |
| Dairy products and eggs | | |
| Yogurt, kefir (kind of buttermilk), sour milk | 2.4 | [1,2,12] |
| Soft-boiled eggs, hard-boiled eggs | 19.9 | [9] |
| Fried eggs, eggs fried in an omelette | 12.4 | [2]** |
| Fresh fruits summer/autumn | | |
| Apple | 0.3 | [1,8,9] |
| Pear | 0.6 | [10] |
| Peach, nectarine | 0.4 | [1,13] |
| Cherries, mirabelle plums, plums | 0.3 | [8,14] |
| Grapes | 1.3 | [10] |
| Strawberries | 0.7 | [1] |
| Other berry fruits (currants, raspberries, blackberries, bilberries) | 4.1 | [13] |
| Bananas | 0.6 | [14] |
| Kiwi fruit | 0.6 | [14] |
| Fresh fruits winter/spring | | |
| Apples | 0.3 | [1,8,9] |
| Oranges, grapefruits | 0.5 | [1,8,10] |
| Bananas | 0.6 | [14] |
| Tangerines, kiwi fruit | 0.6 | [14] |
| Frozen strawberries | 0.7 | [1,15] |
| Beef and Pork | | |
| Beef steak, beef stew | 5.2 | [9,14,16] |
| Roast beef | 6.2 | [1] |
| Boiled beef | 5.5 | [9,16] |
| Pork (pork chops, roast pork, stew) | 11.4 | [9,16] |
| Pork veal | 4.4 | [2,16,17,18,19] |
| Meat and poultry | | |
| Chopped meat, minced meat, schnitzel (roasted paupiette, hamburger, meatball) | 11.4 | [9,16] |
| Liver (pork liver, poultry liver) | 54.2 | [1,2,16] |
| Other organ meats (kidneys, hearts, tripe, lungs) | 26.1 | [16,20] |
| Rabbit | 10.4 | [16,21] |
| Roasted chicken(chicken fillets) | 11.0 | [7,16] |
| Other poultry (chicken leg, duck, turkey) | 12.5 | [7,16] |
| Mutton | 12.7 | [16,22,23] |
| Sausages | | |
| Grilled sausage | 7.2 | [6,16] |
| Weenies, franks, boiled Frankfurter sausage | 14.7 | [6,16] |
| Fish dishes | | |
| Fish (fish fillet, fish sticks) | 12.2 | [16,24] |
| A method of preparing meat and fishes | | |
| Butter | 1.2 | [2] |
| Margarine (margarines such as ‘Benevita’, ‘Apetyczna’, ‘Marcysia’, ‘Ekstra Pomorski’) | 5.3 | [5] |
| Vegetable fat (‘Benevita’ margarine) | 3.3 | [5] |
| Animal fat (lard, backfat) | 6.9 | [25] |
| Olive oil, vegetable oil | 0.1 | [26] |
| Salads | | |
| Lettuce | 2.4 | [2,7] |
| Mixed salad (cucumbers, tomatoes, pepper) | 0.2 | [2,7,13]* |
| Cucumber salad | 5.0 | [2,7]* |
| Carrot salad | 2.9 | [1,2,7,8,9]* |
| Cabbage salad (but not pickled cabbage) | 4.9 | [2,7,26,27]* |
| Radish salad | 4.1 | [1,2,7,27]* |
| Pepper | 1.1 | [7] |
| Raw tomatoes | 1.5 | [7] |
| Chive, onion | 1.7 | [2,7,27] |
| Salad additives | | |
| Olive oil | 0.1 | [26] |
| Other types of oil (i.e. soybean oil) | 0.8 | [10] |
| Cream | 1.5 | [7] |
| Premade dressing, mayomaise | 4.6 | [5,9,26,28]* |
| Vinegar | <0.1 | [28] |
| Lemon juice | 0.7 | [29] |
| Onion (raw) | 1.7 | [2,7,27] |
| Fresh culinary herbs (thyme, coriander, basil, oregano, rosemary, parsley) | 1.9 | [2,30] |
| Cooked vegetables | | |
| Boiled white and red cabbage | 3.8 | [2,3,7,27]** |
| Cauliflower | 6.6 | [2,5,31]** |
| Brussels sprouts | 8.1 | [2,5,31]** |
| Boiled spinach | 1.2 | [2,3,24,32]** |
| Boiled carrot | 2.0 | [2,3]** |
| Cooked beet and other cooked vegetables (i.e. cooked celery) | 0.6 | [3,5,33,34]** |
| Vegetable salad consists of cooked vegetables | 2.7 | [2,5,7,9,13,18,26,27,33]** |
| Green bean, green peas | 16.0 | [2,18,31]** |
| Pickled/Marinated vegetables | | |
| Sauerkraut | 1.8 | [33,35,36] |
| Mushroom dishes | 3.9 | [2]*** |
| Pickled cucumbers and other pickled vegetables (i.e. onion, pepper) | 0.9 | [33] |
| Potatoes and dumplings | | |
| Boiled potatoes | 0.2 | [13,23]*** |
| Mashed potatoes | 0.3 | [2,7,13,23,27]** |
| Fried potatoes, french fries, baked potatoes | 0.3 | [13,23]*** |
| Potato dumplings | 0.3 | [2,3,7,13,23,27]** |
| Dishes of flour | | |
| Pasta | 7.9 | [3]*** |
| Rice | 7.0 | [1,37] |
| Kasha, corn | 3.2 | [1]*** |
| Other dishes | | |
| Pizza | 3.3 | [1,2,3,6,7,26,27,38,39]** |
| Potato cakes | 1.0 | [2,3,7,13,26,27]** |
| Pancakes, croquettes | 5.7 | [2,3,7,27]** |
| Desserts |  |  |
| Canned fruits (from compote) (pineapple, peach) | 0.4 | [40] |
| Cakes | | |
| Layer cake (chocolate torte) | 5.8 | [2,3,5,7,8,10,41,42,43,44,45,46]* |
| Fruit pie | 7.5 | [1,2,3,6]* |
| Whipped cream | 1.3 | [47] |
| Candies and snacks | | |
| Chocolate | 0.4 | [41] |
| Chocolate bars | 0.4 | [48] |
| Cookies (tea cakes), biscuits | 0.4 | [48] |
| Chocolates (i.e. with vodka or other small chocolates) | 0.3 | [48] |
| Ice cream | 10.0 | [49] |
| Chips, salty sticks, crackers | 0.4 | [48] |
| Nuts (i.e. peanuts, hazelnuts) | 9.5 | [14] |
| Coffee and tea | | |
| Coffee | 0.1 | [8] |
| Decaf | 0.1 | [50,51] |
| Chicory coffee | 3.5 | [52] |
| Black tea | 0.1 | [8] |
| Refreshing drinks | | |
| Mineral water, spring water | 0.1 | [41,53] |
| Fruit juices | 0.1 | [54] |
| Multivitamin juice | 0.1 | [29] |
| Lemonade, orangeade | 0.3 | [8,55] |
| Coca-cola, pepsi-cola | 0.3 | [41] |
| Spirits | | |
| Beer | 0.6 | [53,56] |
| Wine | 0.1 | [2,53,57] |
| Fruit wine | 0.2 | [2] |
| Champagne, sparkling wine | 0.2 | [58] |
| Vodka, whisky, cognac, rum | 0.0 | [42] |

* standard recipe (references no 59, 60, 62), selenium content for ingredients,

**standard recipe (references no 59, 61), selenium content for ingredients included losses of selenium due to various cooking practices

***selenium content in single food additives (mushroom, potatoes, pasta) added to main dishes included losses of selenium due to heat treatment

reference no 16 - selenium losses in foods and dishes due to different cooking practices (boiling, canning, frying, grilling)

References to the Appendix

1. Wojtasik A, Pietraś E. Co warto wiedzieć o selenie? [What is worth knowing about selenium?]. In: Website of National Center for Nutrition Education, National Institute of Hygiene. 2020. https://ncez.pl/abc-zywienia-/zasady-zdrowego-zywienia/co-warto-wiedziec-o-selenie-. Polish. Accessed 14 Feb 2020 (temporary access: Jan 2020-Dec 2020).

2. Kuczyńska J, Biziuk M. Biogeochemia selenu i jego monitoring w materiałach biologicznych pochodzenia ludzkiego [Selenium biogeochemistry and its monitoring in biological samples]. Ecol Chem Eng. 2007;14:47-65. Polish.

3. Marzec Z. Produkty zbożowe jako źródło selenu w krajowych racjach pokarmowych [Cereal products as a source of selenium in domestic food rations]. Rocz Panstw Zakl Hig. 2002;53:377-83. Polish.

4. Zawartość selenu we francuskiej bagietce [Selenium content in French baguette]. http://www.ja-i-ty.pl/zdrowie/wartosc-odzywcza/bagietka-francuska.html#mineraly. Polish. Accessed 6 Jul 2021.

5. Read-Gene. Zawartość selenu w pochodzących z rynku polskiego wybranych produktach spożywczych. W: Opracowanie firmy Read-Gene [Selenium content in selected food products from the Polish market. In: Study by Read-Gene]. 2011. http://selenowanie.pl/jadlospisy/zawartosc-selenu-w-produktach/. Polish. Accessed 6 Jul 2021.

6. Read-Gene. Lista produktów ze zmierzonym poziomem selenu. W: Opracowanie firmy Read-Gene [List of products with the measured selenium level. In: Study by Read-Gene]. 2011. http://selenowanie.pl/selen-w-zywnosci/. Polish. Accessed 6 Jul 2021.

7. Smrkolj P, Pograjc L, Hlastan-Ribič C, Stibilj V. Selenium content in selected Slovenian foodstuffs and estimated daily intakes of selenium. Food Chem. 2005;90:691-97.

8. Kadrabova J, Madaric A, Ginter E. The selenium content of selected food from the Slovak Republic. Food Chem. 1997;58:29-32.

9. Oster O, Prellwitz W. The daily dietary selenium intake of West German adults. Biol Trace Elem Res. 1989;20:1-14.

10. Şlencu B, Ciobanu C, Cuciureanu R. Selenium content in foodstuffs and its nutritional requirement for humans. Clujul Med. 2012;85:139-45.

11. Polak-Juszczak L. Składniki mineralne w przetworzonej żywności pochodzenia morskiego [The mineral elements in processed food of marine origin]. Inż Rol. 2007; 5:331-7. Polish.

12. Pilarczyk B, Tomza-Marciniak A, Mituniewicz-Małek A, Wieczorek-Dąbrowska M, Pilarczyk R, Wójcik J, et al. Selenium content in selected products of animal origin and estimation of the degree of cover daily Se requirement in Poland. Int J Food Sci Technol. 2010;45:186-91.

13. Nikonorow M, Urbanek-Karłowska B. Toksykologia żywności [Food toxicology]. 2nd ed. Warszawa: PZWL; 1987. Polish.

14. Jabłońska E, Gromadzińska J, Bertrandt J, Kłos A, Darago A, Wąsowicz W. Zawartość selenu w wybranych artykułach żywnościowych pochodzących z Polski Centralnej [Selenium content in selected food products from central Poland]. Żyw Człow Metab. 2007;34:1440-5. Polish.

15. Gonçalves GAS, Resende NS, Carvalho EEN, Resende JV, Vilas Boas EVB. Effect of pasteurisation and freezing method on bioactive compounds and antioxidant activity of strawberry pulp. Int J Food Sci Nutr. 2017;68(6):682-94.

16. Bratakos MS, Zafiropoulos TF, Siskos PA, Ioannou PV. Selenium losses on cooking Greek foods. Int J Food Sci Technol. 1988;23:585-90.

17. Markiewicz R, Borawska MH. Mięso jako źródło selenu w diecie mieszkańców Podlasia [Meat as a source of selenium in the diet of the inhabitants of Podlasie Region]. Bromat Chem Toksykol. 2005;38:249-52. Polish.

18. Markiewicz-Żukowska R. Selen w żywności. XVI Sympozjum „Środowisko – zdrowie – żywność” 17-19.05.2010 Wykład. 2010. [Selenium in food. XVI Symposium ,,Environment-health-food” 17-19.05.2010 Lecture materials. 2010.] https://www.msspektrum.pl/sympozja/pdfy/slesin2010_borawska_markiewicz_zukowska2.pdf. Polish. Accessed 6 Jul 2021.

19. ABC Zdrowie. Zawartość selenu w cielęcinie [Selenium content in veal]. https://zywienie.abczdrowie.pl/surowa-cielecina-mieso-i-tluszcz. Polish. Accessed 6 Jul 2021.

20. ABC Zdrowie. Zawartość selenu we flaczkach wieprzowych [Selenium content in pork tripe]. https://zywienie.abczdrowie.pl/flaczki-wieprzowe-gotowane-na-wolnym-ogniu. Polish. Accessed 6 Jul 2021.

21. Dalle Zotte A, Szendro Z. The role of rabbit meat as functional food. Meat Sci. 2011;88(3):319-31.

22. Williams P. Nutritional composition of red meat. Nutr Diet. 2007;64:113–19.

23. Morris VC, Levander OA. Selenium content of foods. J Nutr. 1970;100(12):1383-8.

24. Jabłońska E, Gromadzińska J, Kłos A, Bertrandt J, Skibniewska K, Darago A, et al. Selenium, zinc and copper in the Polish diet. J Food Compos Anal. 2013;31:259-65.

25. FITATU. Zawartość selenu w słoninie [Selenium content in backfat]. https://www.fitatu.com/catalog/pl/slonina--1663. Polish. Accessed 6 Jul 2021.

26. Pappa EC, Pappas AC, Surai PF. Selenium content in selected foods from the Greek market and estimation of the daily intake. Sci Total Environ. 2006;372(1):100-8.

27. Kápolna E, Fodor P. Bioavailability of selenium from selenium-enriched green onions (Allium fistulosum) and chives (Allium schoenoprasum) after 'in vitro' gastrointestinal digestion. Int J Food Sci Nutr. 2007;58(4):282-96.

28. Choi Y, Kim J, Lee HS, Kim C, Hwang IK, Park HK, et al. Selenium content in representative Korean foods. J Food Compos Anal. 2009;22:117–22.

29. Gawłoska-Kamocka A. The determination of content of selenium in natural fruit juices by spectral methods. Rocz Panstw Zakl Hig. 2008;59(2):173-8.

30. Ozcan M. Mineral contents of some plants used as condiments in Turkey. Food Chem. 2004;84:437-40.

31. ABC Zdrowie. Zawartość selenu w bułce tartej [Selenium content in bread crumbs]. https://zywienie.abczdrowie.pl/bulka-tarta. Polish. Accessed 6 Jul 2021.

32. ODŻYWIANIE INFO. Zawartość selenu w szpinaku [Amount of selenium in spinach]. https://www.odzywianie.info.pl/przydatne-informacje/artykuly/art,szpinak-kalorie-wartosci-odzywcze-i-ciekawostki.html. Polish. Accessed 6 Jul 2021.

33. Klapec T, Mandić ML, Grgić J, Primorac LG, Perl A, Krstanović V. Selenium in selected foods grown or purchased in eastern Croatia. Food Chem. 2004;85:445–52.

34. Nowak L, Kucharzewski A. Zawartość arsenu i selenu w produktach roślinnych pochodzących z województwa legnickiego [Contents of arsenic and selenium in plant products originated from Legnica province]. Zesz Probl Post Nauk Rol. 2000;471:1067-74. Polish.

35. Traditional oven. Selenium content in sauerkraut. http://www.traditionaloven.com/foods/specific-nutrient/veggies/sauerkraut-can-low-na/selenium-se.html. Accessed 6 Jul 2021.

36. SELF Nutrition Data. Selenium content in sauerkraut. https://nutritiondata.self.com/facts/vegetables-and-vegetableproducts/2614/2. Accessed 6 Jul 2021.

37. Higgs DJ, Morris VC, Levander OA. Effect of cooking on selenium content of foods. J Agric Food Chem. 1972;20(3):678-80.

38. Masłowska J, Janiak J. Poziom molibdenu i selenu w drożdżach piekarskich [Molybdenum and selenium levels in baker’s yeast]. Przegląd Piek i Cukier. 1996;44(05): 12-13. Polish.

39. Bratakos MS, Zafiropoulos TF, Siskos PA, Ioannou PV. Total selenium concentration in tap and bottled drinking water and coastal waters of Greece. Sci Total Environ. 1988;76(1):49-54.

40. Finley J, Matthys L, Schuler T, Korynta E. Selenium content of foods purchased in North Dakota. Nutr Res. 1996;16(5):723–28.

41. Gawłoska – Kamocka A. Selen w artykułach spożywczych. Część 1: Napoje. Część 2: Produkty cukiernicze i zbożowe [Selenium in food products. Part 1: Beverages. Part 2: Confectionery and cereal products]. J Elementol. 2006;11(4):431-37 (part 1), 439-447 (part 2). Polish.

42. Traditional oven. Selenium content in alcoholic beverages: rum, vodka, whisky. http://www.traditionaloven.com/foods/specific-nutrient/beverage/alcoholic-beverage-distilled-all-gin-rum-vodka-whiskey-eighty-six-proof/selenium-se.html. Accessed 6 Jul 2021.

43. Dico GML, Galvano F, Dugo G, D'ascenzi C, Macaluso A, Vella A, et al. Toxic metal levels in cocoa powder and chocolate by ICP-MS method after microwave-assisted digestion. Food Chem. 2018;245:1163-8.

44. ABC Zdrowie. Zawartość selenu w proszku do pieczenia [Selenium content in baking powder]. https://zywienie.abczdrowie.pl/proszek-do-pieczenia. Polish. Accessed 6 Jul 2021.

45. ABC Zdrowie. Zawartość selenu w cukrze pudrze [Selenium content in powdered sugar]. https://zywienie.abczdrowie.pl/cukier-puder. Polish. Accessed 6 Jul 2021.

46. Ekogram. Zawartość selenu w sodzie oczyszczonej [Selenium content in bicarbonate of soda]. https://ekogram.pl/pl/srodki-czystosci/131-soda-oczyszczona-spozywcza-1kg-anagram-zielonki.html. Polish. Accessed 6 Jul 2021.

47. Eat this much. Selenium content in whipped cream. https://www.eatthismuch.com/food/nutrition/whipped-cream,53/. Accessed 6 Jul 2021.

48. Gawłoska - Kamocka A. Speciation analysis of inorganic forms of selenium in confectionery products. J Elementol. 2007;12(2):105-10.

49. Cabrera C, Lorenzo ML, De Mena C, Lopez MC. Chromium, copper, iron, manganese, selenium and zinc levels in dairy products: in vitro study of absorbable fractions. Int J Food Sci Nutr. 1996;47(4):331-9.

50. ABC Zdrowie. Zawartość selenu w kawie bezkofeinowej [Selenium content in decaffeinated coffee]. https://zywienie.abczdrowie.pl/bezkofeinowa-kawa-instant-przygotowana-z-woda. Polish. Accessed 6 Jul 2021.

51. Diet and Fitness today. Amount of selenium in coffee. http://www.dietandfitnesstoday.com/selenium-in-coffee.php. Accessed 6 Jul 2021.

52. Rybicka I, Krawczyk M, Stanisz E, Gliszczyńska-Świgło A. Selenium in gluten-free products. Plant Foods Hum Nutr. 2015;70(2):128-34.

53. Ulewicz Magulska M. Selen w roślinnych surowcach leczniczych, zawartość, rozmieszczenie i wzajemne relacje z innymi pierwiastkami. Praca doktorska wykonana w Akademii Medycznej w Gdańsku. 2008. [Selenium in medicinal plant raw materials, content, distribution and interplay between selenium and other elements. PhD Thesis, Medical Academy in Gdańsk. 2008.]. Polish.

54. Masłowska J, Gawłoska A. Spektralne metody oznaczania śladowych ilości selenu w wodach naturalnych oraz sokach owocowych [A comparison of spectral methods for the determination of trace quantities of selenium in natural drinking water and fruit juice]. Bromat Chem Toksykol. 2000;1:91-7. Polish.

55. Diet and Fitness today. Amount of selenium in lemonade. http://www.dietandfitnesstoday.com/selenium-in-lemonade.php. Accessed 6 Jul 2021.

56. Kordialik-Bogacka E. Wartość żywieniowa piwa [Nutritional value of beer]. In: Walczycka M, Duda-Chodak A, Jaworska G, Tarko T, editors. Żywność projektowana. Kraków: Oddział Małopolski Polskiego Towarzystwa Technologów Żywności; 2011. p. 124-134. Polish.

57. Niesteruk R. Właściwości termofizyczne żywności. Część II [Thermophysical properties of food. Part 2]. Białystok: Wydawnictwa Politechniki Białostockiej; 1999. Polish.

58. Diet and Fitness today. Selenium content in alcoholic beverages. http://www.dietandfitnesstoday.com/selenium-in-alcohol.php. Polish. Accessed 6 Jul 2021.

59. Łukasiak J, Ostrowska H. Książka kucharska [Recipe book]. 7th ed. Warszawa: Wydawnictwo spółdzielcze; 1990. Polish.

60. Hello Tesco. Przepis na majonez [Recipe for mayonnaise]. https://www.tesco.pl/smaczna-strona/porady/jak-zrobic-domowy-majonez-178.html. Polish. Accessed 6 Jul 2021.

61. Aromatyczne inspiracje. Przepis na pizzę [Recipe for preparing pizza]. http://aromatyczneinspiracje.blogspot.com/2012/02/pizza-z-szynka-pieczarkami-i-kukurydza.html. Polish. Accessed 6 Jul 2021.

62. Kwestia smaku. Przepis na tort [Recipe for preparing chocolate layer cake]. http://www.kwestiasmaku.com/przepis/torcik-czekoladowy-przekladany. Polish. Accessed 6 Jul 2021.

Appendix 2. Controls’ diagnoses by ICD-X codes

| ICD-X | number |
| --- | --- |
| D53 | 1 |
| D64 | 2 |
| D67 | 1 |
| D69 | 1 |
| D73 | 1 |
| E01 | 1 |
| E03 | 1 |
| E04 | 16 |
| E05 | 1 |
| E06 | 1 |
| E27 | 1 |
| EOS | 1 |
| F32 | 1 |
| I10 | 82 |
| I11 | 13 |
| I13 | 1 |
| I20 | 84 |
| I21 | 30 |
| I24 | 5 |
| I25 | 44 |
| I34 | 1 |
| I35 | 3 |
| I45 | 1 |
| I47 | 1 |
| I48 | 24 |
| I49 | 2 |
| I50 | 1 |
| I65 | 1 |
| I70 | 1 |
| I71 | 1 |
| I73 | 2 |
| I74 | 2 |
| I8 | 1 |
| I80 | 4 |
| I84 | 3 |
| I99 | 1 |
| J18 | 29 |
| J20 | 3 |
| J41 | 9 |
| J42 | 5 |
| J44 | 9 |
| J45 | 9 |
| J85 | 1 |
| K21 | 1 |
| K35 | 1 |
| K40 | 46 |
| K42 | 16 |
| K43 | 7 |
| K44 | 3 |
| K45 | 4 |
| K46 | 3 |
| K60 | 3 |
| K76 | 1 |
| K80 | 122 |
| K81 | 5 |
| K82 | 5 |
| K83 | 11 |
| K92 | 1 |
| M13 | 1 |
| M19 | 1 |
| M47 | 1 |
| M62 | 1 |
| M86 | 1 |
| N10 | 3 |
| N11 | 2 |
| N12 | 1 |
| N20 | 1 |
| N23 | 1 |
| N93 | 1 |
| Q44 | 1 |
| R00 | 1 |
| R02 | 1 |
| R07 | 13 |
| R09 | 1 |
| R10 | 1 |
| R39 | 1 |
| R50 | 5 |
| R51 | 1 |
| R55 | 18 |
| R57 | 1 |
| R59 | 1 |
| R60 | 1 |
| S00 | 3 |
| S01 | 2 |
| S02 | 2 |
| S22 | 2 |
| S32 | 1 |
| S42 | 4 |
| S72 | 4 |
| S82 | 7 |
| S83 | 3 |
| S89 | 3 |
| S90 | 1 |
| S93 | 2 |
| T23 | 1 |
| T78 | 2 |
| W86 | 1 |
| missing | 30 |
| Total |  |
